# Real-World Treatment Patterns and Outcomes of Growth Hormone Treatment Among Children in Israel Over the Past Decade (2004–2015)

**DOI:** 10.3389/fped.2021.711979

**Published:** 2021-08-20

**Authors:** Tal Ben-Ari, Gabriel Chodick, Varda Shalev, Dalit Goldstein, Roy Gomez, Zohar Landau

**Affiliations:** ^1^MaccabiTech, Maccabi Healthcare Services, Tel Aviv, Israel; ^2^Sackler Faculty of Medicine, Tel Aviv University, Tel Aviv, Israel; ^3^Pediatric Endocrinology Unit, Edith Wolfson Medical Center, Holon, Israel; ^4^Global Medical Affairs, Pfizer Rare Disease, Brussels, Belgium; ^5^Pediatric Division, Barzilai Medical Center, Ashkelon, Israel; ^6^Faculty of Health Sciences, Ben-Gurion University of the Negev, Beer-Sheva, Israel

**Keywords:** children, growth, growth hormone, growth hormone deficiency, height, idiopathic short stature

## Abstract

**Objective:** To assess a decade of growth hormone (GH) treatment patterns and outcomes in a real-world setting in Israel using a state-of-the-art computerized database.

**Methods:** This large retrospective database study included 2,379 children initiating GH treatment in Maccabi Healthcare Services (between January 2004 and December 2014). Good adherence with therapy (proportion of days covered >80%) was assessed during follow-up.

**Results:** At GH treatment initiation: 62.1% were boys; height standard deviation score (SDS) was −2.36 ± 0.65 (mean ± SD); age was 9.8 ± 3.1 years; and time from short stature diagnosis to first GH purchase was 4.8 ± 3.3 years. Mean treatment period was 3.5 ± 0.95 years; 79.4% of children were treated for more than 3 years. The two main indications for GH therapy were idiopathic short stature (ISS) (*n* = 1,615, 67.9%) and GH deficiency (GHD) (*n* = 611, 25.7%). Children in the highest socio-economic-status (SES) tertile comprised 61.3% of ISS and 59.7% of GHD. After 3 years, mean height gain SDS was 1.09 ± 0.91 for GHD and 0.96 ± 0.57 for ISS (*p* = 0.0004). Adult height (age 15 for girls and 17 for boys) was recorded for 624 patients (26.2%) with better outcomes for GHD than ISS (−1.0±0.82 vs. −1.28±0.93, respectively; *p* = 0.0002). Good adherence was achieved in 78.2% of the cohort during the first year and declined thereafter to 68.1% during the third year of the treatment.

**Conclusions:** Children who initiate GH therapy are predominantly male, belong mainly to the upper SES, commence treatment a long period after initial recognition of short stature, and have suboptimal adherence. Appropriate referral, diagnosis, and follow-up care may result in better treatment outcomes with GH therapy.

## Introduction

Growth hormone (GH) has been the standard therapy for over 60 years for the treatment of children with growth disorders (1), and since 1985 in the form of recombinant human growth hormone (rhGH, somatropin) (2). The objectives of pediatric GH therapy during childhood and adolescence are to normalize height velocity as quickly as possible and attain a final adult height within the normal range, while minimizing risks and cost.

The efficacy and safety profiles of GH therapy have led to the approval of GH use in many countries for the treatment of growth disorders, including GH deficiency (GHD) (3), idiopathic short stature (ISS) (3), Turner syndrome (TS), Prader-Willi syndrome (PWS), chronic renal failure (CRF), small for gestational age (SGA), and Noonan's Syndrome (4, 5).

The two main pediatric indications for GH therapy are GHD and ISS (3). A diagnosis of GHD in a child is characterized by a subnormal growth rate and failure to respond to at least two GH stimulation tests, among other criteria (6, 7). Children with ISS have a height standard deviation score (SDS) that is more than two SDs below the mean for normal peers without evidence of other disease processes, and have stimulated serum GH levels within the normal range (8).

Despite global short-term evidence for GH therapy, limited long-term data are available on treatment patterns and outcomes of GH use. The available evidence suggests that success of GH therapy depends on a number of factors including age at initiation of treatment (6), adherence (9), indication, and gender (10). Most of the current information regarding treatment patterns and long-term outcome of GH therapy is derived from pharmaceutical based registries [Kabi International Growth Study (KIGS), NordiNet® International Outcome Study, ANSWER] and several nationwide cohorts (11–14), which provide limited data on the baseline characteristics at GH initiation by indication, outcome, and adherence in a real-world environment.

The increasing availability of large electronic databases maintained by hospitals, Health Maintenance Organizations (HMOs), and other health service providers potentially offers a richer source of information regarding GH treatment patterns and outcomes. Maccabi Healthcare Services (MHS) is Israel's second largest state-mandated healthcare provider, with 2.5 million members. MHS maintains a comprehensive state-of-the-art computerized database that includes 20 years of demographic and medical data on its members (15, 16).

The MHS presents a unique opportunity to study GH treatment patterns in children in a real-world setting, from GH treatment initiation to attainment of adult height.

The objectives of this retrospective study were to evaluate prescribing patterns, adherence, and outcomes of GH treatment in a large cohort of pediatric patients in Israel, mainly focusing on the GHD and ISS indications. The patients were followed from treatment initiation of GH over a period of at least 12 months from 2004 to 2015. The MHS records clinical data including growth parameters, the indication for treatment, and demographic data including socioeconomic factors, all of which were evaluated in this study.

## Methods

### Study Design

A retrospective database analysis of a cohort of pediatric patients treated with GH for growth disorders between January 1, 2004 and December 31, 2014 in Israel, was conducted using patients identified from the MHS database. The MHS database, which includes up to 20 years of data on 2 million members, represents a sample comprising 25% of the Israeli population (16). The database integrates data from the patients' electronic medical records (EMR) that include anthropometric measurements, MHS central laboratory, medication prescriptions and their indications, purchases throughout the MHS pharmacy network, consultations, hospitalizations, procedures, and sociodemographic data (17). Children's treatment patterns and outcomes from GH treatment initiation were assessed over a follow-up period of at least 12 months in this study.

### Study Population

The study population comprised all children who were up to 18 years of age at initiation of GH treatment with at least one purchase of GH during 2004 to 2014 and were treated with GH for GHD, ISS, TS, PWS, CRF, SGA, and Noonan's Syndrome. Additional inclusion criteria were: continuous enrolment in MHS for at least 1 year prior to GH initiation; baseline height recorded prior to GH initiation; and at least one follow-up height measurement within 2 years after GH treatment initiation. Subjects were excluded if they had <12 months of follow-up from GH initiation (e.g., left the MHS database), and diagnosis of severe disease (cancer, end stage renal failure) at baseline or within 1 year of GH initiation. GH deficiency was defined according to the guidelines published by the Israeli Society of Pediatric Endocrinology as a peak serum GH <7.5 ng/ml in response to two pharmacological GH provocative tests (clonidine, arginine, or glucagon). Girls above the age of 11 years were primed with oestradiol and boys above the age of 13 years were primed with testosterone. This study was approved by the MHS ethics committee. Because there was no identification of the subjects for whom data were retrieved, informed consent from the parents was waived.

### Data

Data included anthropometric measures (weight and height), information on medication prescriptions, indication, and purchases throughout the MHS pharmacy network, and socioeconomic data (SES). Height SDS was calculated according to World Health Organization growth charts (actual height—mean height for age/standard deviation [SD] at that age). The SES of individuals were stratified into three categories (low, intermediate, or high), according to geocoding techniques, by linking the address of the patient's residence with the Census area-level SES data, based on the ratings of Israel's Central Bureau of Statistics (18).

GH therapy indication was coded using the International Classification of Diseases (9th Edition [ICD-9-CM]) in addition to internal MHS codes for sub-classification. In MHS, GH treatment requires individual approval conducted by MHS pharmacy approvals center. All GH users and their indication to treatment were documented in the approval center, and had to go through the same approval criteria. If a patient had more than one indication for GH treatment, the earliest indication documented in the pharmacy approvals center was recorded.

### Follow-Up Period

The “index date” represented the **first** purchase of GH treatment, between January 1, 2004 and December 31, 2014. Time between first diagnosis to first GH purchase was calculated from first documented short stature diagnosis in patient's EMR. The baseline period was 12 months prior index date. All measurements obtained for this period were the closest to the index date.

The study period for each patient began 12 months prior to the index date and continued until the patient left the MHS or reached the end of the study period (December 31, 2015).

### Baseline Variables

All measurements obtained for this period were the most recent prior to index date. The closest height measurement before index date was defined as baseline height, while follow-up height was the measurement per year of follow-up. Adult height was defined as height achieved in girls after the age of 15 years and in boys after the age of 17 years. Patients who did not reach these ages within the study period were excluded from adult height analysis.

Persistence with therapy was calculated in months passed since the first GH purchase to the latest GH purchase plus the days of the latest prescription. Discontinuation with treatment was defined as a treatment gap of 365 days or longer.

Adherence with GH therapy was estimated using proportion of days covered (PDC) with GH during the follow-up period. PDC is calculated as the ratio of the number of days a patient purchased and retrieved GH treatment at the MHC pharmacies (quantity of vials and pens purchased × length of time each should last) to the number of days they were prescribed GH treatment during that period by their physician. Data for prescriptions and purchases was retrieved from the MHC database. A PDC above 80% suggests that a patient is highly adherent (19).

### Statistical Analysis

Descriptive statistics were presented as n, %, or mean ± SD. Differences between groups were tested using *t*-tests and χ^2^ tests. Persistence with therapy was calculated using Kaplan-Meier survival analysis. Changes in blood assessments were compared using Mann-Whitney U test. All analyses were performed using IBM SPSS v.24® (IBM, New York, United States).

## Results

### Baseline Patient Characteristics

**Figure 1:**
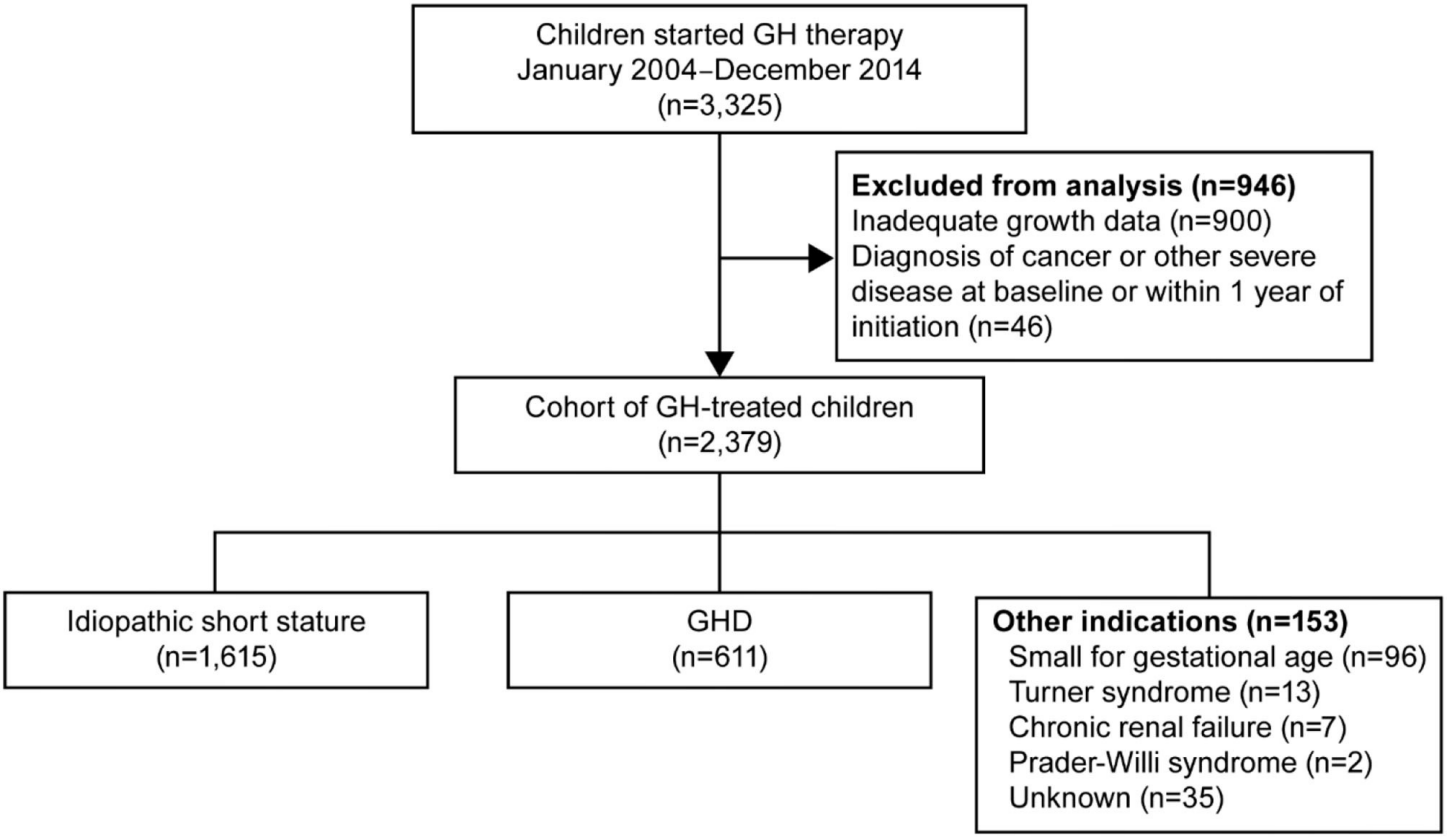
Study flow diagram. GH, growth hormone.

The analysis included 3,325 children (age 0–18 years) over the 2004–2014 time period who initiated treatment with GH. Of these, 946 were excluded from the data analysis for various reasons (Figure 1). The excluded patients were significantly younger at initiation of treatment than the patients included in the analysis (9.8 ± 3.1 years vs. 10.5 ± 8.3 years, respectively; *p* = 0.0004). Excluded patients were also taller than the patients included (height SDS −2.36 ± 0.65 vs. −2.27 ± 1.13, respectively; *p* = 0.0039). Gender, SES, and prevalence of indications for GH therapy were similar in the excluded and analysis groups. The evaluated cohort comprised 2,379 patients treated with GH. The two main indications for GH therapy were ISS (*n* = 1,615, 67.9%) and GHD (*n* = 611, 25.7%). A total of 153 patients were treated for other indications (Figure 1).

The data from the patients with ISS and GHD (*n* = 2,226) are focused on in detail hereafter. The clinical characteristics at baseline of patients in each of the ISS and GHD diagnostic cohorts are summarized in Table 1.

**Table 1:** Baseline characteristics and treatment patterns.

	**ISS (***n*** = 1,615)**	**GHD (***n*** = 611)**	***P*** **-value**
Gender			
Male	1,006 (62.3%)	383 (62.7%)	0.871
Age at initiation (years)	10.12 (±2.9)	9.42 (±3.42)	0.001
<5	73 (4.5%)	89 (14.6%)	<0.001
5–9	683 (42.3%)	223 (36.5%)	<0.001
10–14	824 (51.0%)	281 (46.0%)	0.035
15–17	35 (2.2%)	18 (2.9 %)	0.33
SES			
Low	76 (4.7%)	29 (4.8%)	0.92
Intermediate	548 (34.0%)	217 (35.6%)	0.47
High	989 (61.3%)	364 (59.7%)	0.49
Weight SDS at baseline	−0.98 (±0.54)	−1.10 (±0.86)	0.001
Height SDS at baseline	−2.36 (±0.60)	−2.30 (±0.67)	−0.042
BMI SDS at baseline	0.51 (±0.23)	0.29 (±0.68)	<0.001
Time from diagnosis of short stature in EMR to GH treatment initiation (years)	5.0 (±3.3)	4.4 (±3.1)	0.001
Persistence with treatment (years)	3.40 (±1.91)	3.61 (±1.68)	0.017
Highly adherent (PDC >80%)			
Overall	948 (58.7%)	411 (67.3%)	<0.001
Year 1	1,226 (75.9%)	512 (83.8%)	<0.001
Year 2	889 (55.0%)	407 (66.6%)	<0.001
Year 3	1,042 (64.5%)	473 (77.4%)	<0.001
Discontinuation *n* (%)	84 (5.2)	23 (3.7)	0.15

*Values are number (%) or mean (SD) as appropriate*.

*EMR, electronic medical records; BMI, body mass index; GH, growth hormone; GHD, growth hormone deficiency; ISS, idiopathic short stature; PDC, proportion of days covered; SD, standard deviation; SES, socioeconomic status*.

A similar proportion of patients were male in the GHD (62.7%) and ISS (62.3%) groups. Height SDS at baseline was lower in the ISS group than in the GHD group (−2.36 ± 0.60 and −2.30 ± 0.67, respectively; *p* = 0.042). The height SDS at baseline in the overall study population was −2.36 ± 0.60.

The time between first documented diagnosis of short stature in the EMR to first GH purchases was significantly shorter among children with GHD compared with those with ISS (4.4 ± 3.1 years and 5.0 ± 3.3 years; *p* = 0.001) and children with GHD initiated treatment at a younger age (9.42±3.42 vs. 10.12 ± 2.9 years in GHD and ISS, respectively, *p* = 0.0001) (Table 1). The age of initiation among the overall study cohort was 9.81 ± 3.13 years.

**Figure 2:**
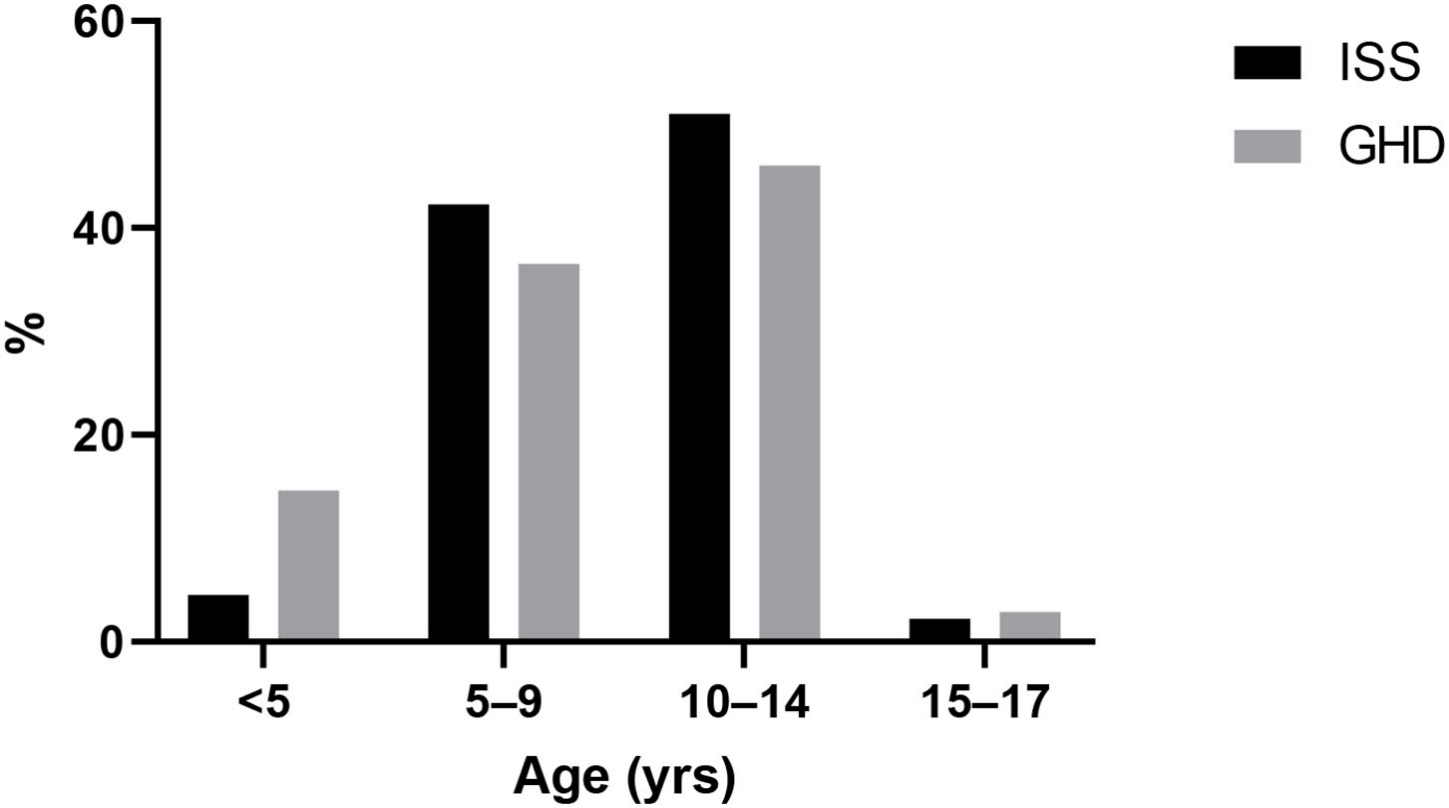
Age at initiation of growth hormone treatment. ISS, idiopathic short stature; GH, growth hormone; GHD, growth hormone deficiency.

Children between the ages of 10 and 14 years comprised the largest age group that initiated GH treatment for both groups, while among the GHD group a higher proportion of children initiated treatment under the age of 5 years (14.6 vs. 4.5% for GHD and ISS, respectively; *p* < 0.0001) as presented in Figure 2.

The proportion of children belonging to the upper third of SES was the largest and was similar for both diagnoses, comprising 59.7 and 61.3% among the GHD and ISS groups, respectively (*p* = 0.49) (Table 1).

### Treatment Patterns

#### Treatment Duration (Persistence)

79.4% (1,890 out of 2,379 GH-treated patients) were treated for a period of at least 3 years during the study period. Mean persistence with treatment of children with GHD was significantly longer compared with children with ISS (3.6 ± 1.6 and 3.4 ± 1.9 years for GHD and ISS, respectively; *p* = 0.017) (Table 1).

#### Adherence and Discontinuation

Adherence, defined as PDC >80%, was achieved in 78.2% of the whole cohort during the first year of the treatment, declined to 57.6% during the second year, and increased to 68.1% during the third year of the treatment. Adherence was significantly better among children with GHD compared with ISS patients over the whole treatment period and from the first year of treatment to the third year of treatment (Table 1). Rates of discontinuation was similar among patients with ISS and GHD (*p* = 0.14).

### Outcomes

#### Height Outcomes

**Figure 3:**
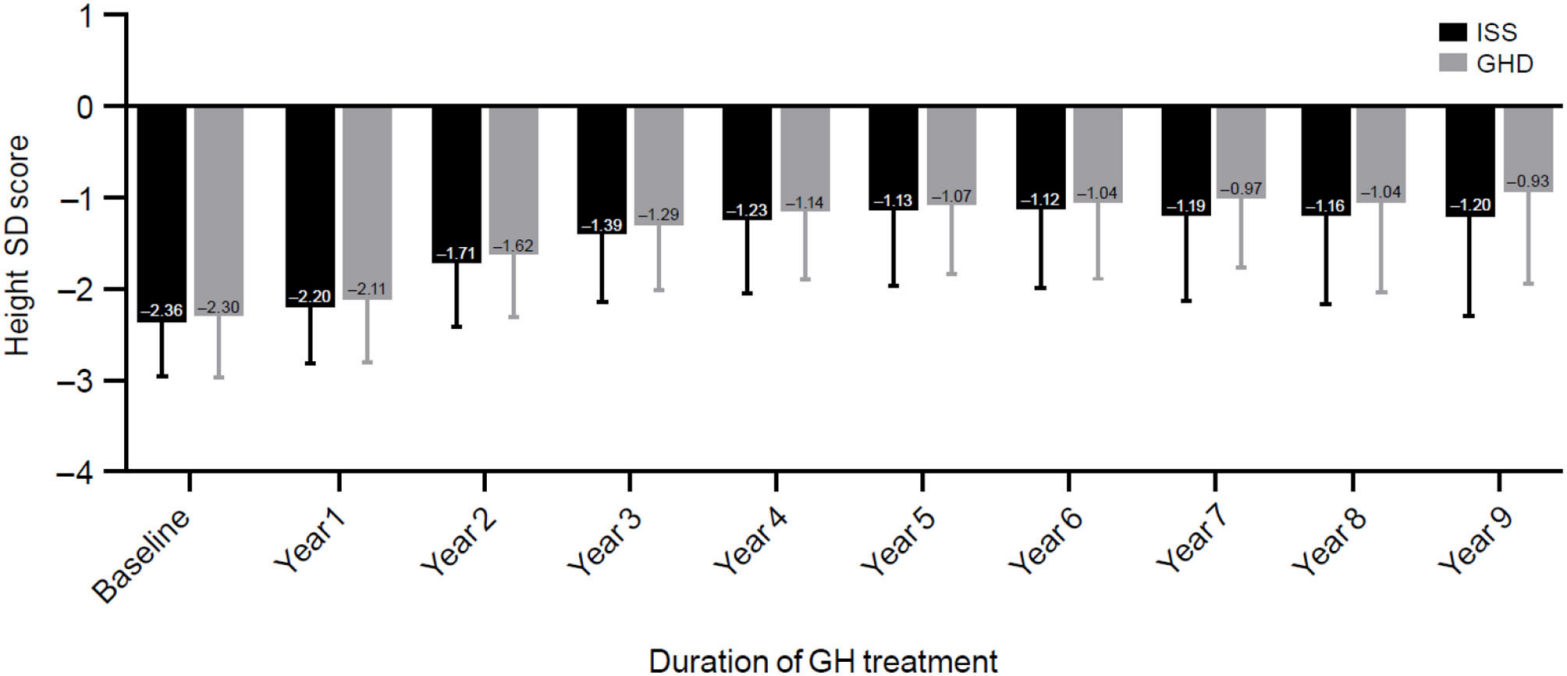
Changes in height standard deviation score. ISS, idiopathic short stature; GH, growth hormone; GHD, growth hormone deficiency, SD, standard deviation; SDS, standard deviation score.

After 3 years of treatment with GH, height SDS improved by 0.96 ± 0.57 (−2.36 to −1.39) in the ISS group and 1.09 ± 0.91 (−2.3 to −1.29) in the GHD group; *p* = 0.001 (Table 2). The change in height SDS among treated patients according to years of treatment and indication is presented in Figure 3. The majority of the height SDS change was achieved in the first 2 years of treatment (Figure 3). Children with GHD achieved a better adult height compared with patients with ISS (−1.0 ± 0.82 for those with GHD vs. −1.28 ± 0.93 for those with ISS, respectively; *p* = 0.0002).

**Table 2:** Change in height standard deviation score from baseline to adult height.

**Height SDS**	**ISS**	**GHD**	***P*** **-value**
Baseline	−2.36 ± 0.6 (*n* = 1,615)	−2.30 ± 0.67 (*n* = 611)	0.042
Year 3	−1.39 ± 0.76 (*n* = 1,296)	−1.29 ± 0.72 (*n* = 473)	0.005
Adult height	−1.28 ± 0.93 (*n* = 497)	−1.0 ± 0.82 (*n* = 127)	0.001
**Change in height SDS**			
Baseline	—	—	
Year 3	0.96 ± 0.57	1.09 ± 0.91	0.001
Adult height	1.05 ± 0.54	1.29 ± 0.68	0.001

*Values are number (%) or mean (SD) as appropriate*.

*GHD, growth hormone deficiency; ISS, idiopathic short stature; SD, standard deviation; SDS, standard deviation score*.

## Discussion

This study used real-world retrospective data to analyze baseline characteristics, treatment patterns, and outcomes of GH therapy in 2,379 children with GHD and ISS in Israel over a follow-up period of at least 12 months. While the short-term efficacy and safety of GH therapy are demonstrated in randomized controlled trials (20), long-term retrospective cohort studies such as this one provide an important balance to clinical trials by providing information on real-world patient characteristics, treatment practices, and outcomes, including follow-up to adult height, which can differ greatly in a controlled trial setting (21).

Several large post-marketing surveillance or registry studies assessing GH therapy have been conducted internationally, including the KIGS, NordiNet, and ANSWER programs, with many interim analyses already published (12, 22, 23). A limitation of these studies is that they rely on voluntary reporting by clinicians in multiple centers, with differences in treatment practices and reporting standards that may lead to bias in data on treatment indications and outcomes.

The large, comprehensive database of the MHS provides a unique opportunity to study GH treatment patterns and outcomes of a large cohort in a real-world setting where all treated children were approved and documented in accordance with uniform criteria (24).

Children with ISS and GHD comprised the majority of the total cohort in this study. Children with ISS represented the largest subgroup (68 vs. 26% for GHD), a significantly higher ratio for ISS than has been reported elsewhere. Of the 83,803 children enrolled in the KIGS database worldwide, approximately 8% were diagnosed with ISS, vs. 57% with GHD (25). In Europe, where ISS is not an approved indication, the ratio of ISS patients is considerably lower than in the United States, where it has been approved by the Food and Drug Administration since 2003 (1% compared with 17%) (12).

Children with ISS constitute the largest population of potential pediatric candidates for GH, with an incidence of 120 per 10,000, compared with 2.86 per 10,000 for GHD (26). However, GH treatment rates for ISS vary between countries. Such variation may be attributed to differences in insurance coverage rules, access to health care, and attitudes toward treating children with ISS by patient families and/or their treating pediatricians and endocrinologists (24, 27).

The average age at initiation of GH treatment in our cohort was 9.8 years with the GHD group initiating treatment approximately a year earlier. Similar mean ages at initiation were reported in the European cohort in the NordiNet study (9.1 years vs. 10.1 years for GHD and ISS, respectively), while children in the United States were approximately 1 year older at the initiation of treatment in the ANSWER study (11.1 years and 11.4 for GHD and ISS, respectively) (12).

It is generally agreed that for optimal efficacy, GH treatment should be initiated as early as possible (23, 28, 29). Children who start GH treatment earlier, for both GHD and ISS indications, have a better chance of reaching their genetic potential vs. those who delay their treatment (12, 22).

In our cohort, the peri-pubertal ages (10–14 years) comprised the largest age group that initiated GH treatment for both GHD and ISS diagnoses. Similarly, in a study of 93,736 patients enrolled in four US pediatric GH registries, Grimberg et al. showed that the median age at GH initiation was 11 years for girls and 12 years for boys (30). In a study of 9,294 patients with GHD enrolled in the NordiNet study, Polak et al. found that 52.3% of patients commenced treatment above the age of 10 years for girls and 11 years for boys (31).

This study found that the average time from first diagnosis of short stature in a child's EMR to the date of treatment initiation was 4.8 years and was longer for patients with ISS than patients with GHD. To our knowledge, this is the first study to report such data. Several factors may contribute to the delay in the initiation of treatment. In order to obtain treatment, children must first be referred by their primary care physician to a pediatric endocrinologist for diagnosis and treatment decision. The process of diagnosis is complex and time-consuming (32). Studies have shown that primary care physicians vary widely in referring children with short stature to endocrinologists. Pediatric endocrinologists vary in decision-making for GH therapy for children who are physiologically alike, and referring physicians and endocrinologists are both influenced by the degree of parental concern (33–35). Parental concerns about the burden of treatment may be greater in younger years, and concerns about the psychological impact of short stature may be greater in the peri-pubertal period, when the approach of puberty promotes growth plate fusion and limits the remaining opportunity for potential medical intervention (30).

The majority of children treated with GH in this study belonged to the highest SES tertile. A similar pattern was observed for the GHD and ISS subgroups. The SES distribution of the cohort significantly differed from that of the general pediatric population in the MHS (where 23% belongs to the highest SES tertile and 14.4% belongs to the lowest SES tertile). This reflects the true GH treatment bias based on the socioeconomic status of the child and his family.

Few studies have analyzed the relationship between SES and GH treatment patterns. Farfel et al. reported that the majority of children treated with GH for more than 2 years under the Clalit HMO in Israel belonged to the lowest SES group (42%), while only 20% belonged to the highest SES group (24). This may be due in part to different demographics of the two HMOs (24).

In Israel, GH treatment is available to all children meeting the criteria for GHD and ISS, and the cost of treatment is largely subsidized by the HMOs. The out-of-pocket expense to patients following reimbursement is ~$100 per month. Therefore, the cost of treatment is unlikely to be the sole cause of the predominance of children from the upper SES tertile. In children with moderate growth impairment (the majority of patients), referral decisions are strongly influenced by the level of parental concern and physician attitudes (35). Higher educational level has been associated with a higher level of parental concern and a greater likelihood to seek evaluation for short stature (30). In a study of 154 children who were evaluated for short stature, Finkelstein et al. found that parents seeking evaluation of their children's short stature had higher income and educational levels than the surrounding population (33).

The primary objectives of GH treatment are acceleration of growth velocity to promote normalization of growth and stature during childhood and attainment of normal adult height appropriate for that child's genetic potential (6). We found that the mean duration of treatment was 3.6 years for both the GHD and ISS groups, with most of the height SDS change achieved in the first 2 years of treatment in both groups, as has been reported in other studies (36).

Almost a third of our cohort reached near-adult height during the study period. Patients with GHD achieved better overall height gain. However, children in both subgroups reached near-adult height within the normal range (within 2SDS of the mean). This is consistent with the results of previous observational studies, showing that children with GHD reach a mean height SDS of approximately −1.0 (6, 23, 37) and children with ISS reach a mean height SDS of approximately −1.4 (29).

Recombinant GH therapy, similar to other chronic long-term treatments, involves daily subcutaneous injections which, as a result of “treatment fatigue” when the load is placed on the child, could negatively influence adherence (38). Non-adherence to therapy may vary from omitting a dose intermittently, to reducing the amount of dose, or omitting doses completely (36). Several methods are used to measure adherence to GH therapy. Here, the PDC was chosen as a proxy for adherence, with advantages that it is an objective measure and the data are easy to obtain from a large database such as the MHS (prescriptions handed by MHC physicians and patient retrievals from MHC pharmacies); however, the method does not take into account failure to administer GH to the child.

A significantly higher proportion of patients with GHD than patients with ISS maintained good adherence during the period of their treatment. There may be a number of reasons for the better adherence of the subgroup with GHD. As noted, a significantly higher proportion of children with GHD initiated treatment under the age of 5 years, making it more likely that parents or family members took responsibility for administering the treatment. Response to GH therapy was also better in patients with GHD, which could contribute to better adherence. Finally, better adherence in children with GHD may reflect an understanding by parents and caregivers of the health benefits other than growth of GH therapy in this group, including cardiac function, lipid metabolism, and bone density.

Previous studies suggest that adherence to GH therapy ranges from 5 to 82% (9, 39). In a large, retrospective Israeli study examining long-term adherence with GH therapy, Farfel et al. reported that only 30% of patients had good adherence (defined as >10 months per year of pharmacy purchase of GH) (24). There are limited published studies regarding adherence trends over time; however, available evidence indicates that a patient's persistence in chronic conditions considerably reduces after 6 months of treatment (40, 41). Here, adherence for both the GHD and ISS groups was highest in the first year of treatment (84 vs. 76% for GHD and ISS, respectively).

Efforts to improve adherence beyond the first year of treatment should be targeted to patients. An e-Health platform to monitor adherence may help to obtain better outcome and treatment with long-acting GH compounds that enable less intensive treatment protocols.

A strength of this study is the large cohort size and the quality of the data evaluated, which was collated from one HMO with a central regulator, which enforces standardized clinical practices and indications for GH treatment. The MHS database allowed meaningful insight into adult height, patient characteristics, and treatment patterns through evaluations of treatment purchasing, persistence, adherence, and time from diagnosis of short stature to treatment initiation of GH. The study had a follow-up duration of at least 12 months and up to 10 years, and was therefore able to provide insight into long-term treatment patterns and outcomes. Further study is required to elucidate the relationship of SES and adherence and adult height achieved.

Several limitations exist in our study that are related to the nature of a retrospective observational study and rely on data from EMR: a large number of patients were excluded from the analyses for having insufficient data. Despite this, it should be noted that the overall sample size remained high. Adult height achievement relies on bone age and growth velocity. As data regarding pubertal status or bone age and parental heights were not available for analysis, the age of 15 years was used in girls and 17 years in boys as the point of near-adult height (23).

## Conclusions

Children typically initiated GH treatment a long period after initial recognition of short stature, with most of them achieving an adult height within the normal range. A significant proportion of children demonstrated suboptimal adherence from the second year of treatment, which was most pronounced in children with ISS compared with children with GHD. Healthcare providers should be aware that GH treatment may be under-recognized in children of lower SES. Appropriate referral, diagnosis, and follow-up care of children may result in better treatment outcomes with GH.

## Data Availability Statement

The anonymised data supporting the conclusions of this article was obtained from the Maccabi health database (MHS) under a collaborative research agreement and as such and as per MHS's policy, the raw Data cannot be shared due to regulatory limitations.

## Ethics Statement

This study was approved by the Maccabi Health Services ethics committee. Written informed consent from the participants' legal guardian/next of kin was not required to participate in this study in accordance with the national legislation and the institutional requirements.

## Author Contributions

TB-A and ZL were involved in contributing to the concept and design, acquisition, analysis, or interpretation of data, statistical analysis, and drafting of the manuscript. All authors discussed the results, were involved in critical revisions of the drafts, and contributed to the final manuscript.

## Conflict of Interest

RG is an employee and/or stockholder of Pfizer. The remaining authors declare that the research was conducted in the absence of any commercial or financial relationships that could be construed as a potential conflict of interest. The authors declare that this study received funding from Pfizer. The funder was involved in study design and preparation of the manuscript for publication. Editorial/medical writing support was provided by Sharmy Blows, PhD, of Engage Scientific Solutions and was funded by Pfizer.

## Publisher's Note

All claims expressed in this article are solely those of the authors and do not necessarily represent those of their affiliated organizations, or those of the publisher, the editors and the reviewers. Any product that may be evaluated in this article, or claim that may be made by its manufacturer, is not guaranteed or endorsed by the publisher.

## Abbreviations

GH: growth hormone
GHD: growth hormone deficiency
ISS: idiopathic short stature
PWS: Prader-Willi syndrome
rhGH: recombinant human growth hormone
SES: socioeconomic status
SGA: small for gestational age
TS: turner syndrome.
